# Pulmonary Thromboembolism following Russell’s Viper Bites

**DOI:** 10.3390/toxins16050222

**Published:** 2024-05-11

**Authors:** Subramanian Senthilkumaran, Sasikumar Sampath, José R. Almeida, Jarred Williams, Harry F. Williams, Ketan Patel, Ponniah Thirumalaikolundusubramanian, Sakthivel Vaiyapuri

**Affiliations:** 1 Manian Medical Centre, Erode 638001, India; maniansenthil76@gmail.com; 2 Primary Health Care Corporation, Doha 26555, Qatar; sasisampath17@gmail.com; 3 School of Pharmacy, University of Reading, Reading RG6 6UB, UK; j.r.dealmeida@reading.ac.uk (J.R.A.); j.williams4@pgr.reading.ac.uk (J.W.); 4 Toxiven Biotech Private Limited, Coimbatore 641042, India; harry@toxiven.com; 5 School of Biological Sciences, University of Reading, Reading RG6 6UB, UK; ketan.patel@reading.ac.uk; 6 The Tamil Nadu Dr M.G.R Medical University, Chennai 600032, India; ponniah.tks@gmail.com

**Keywords:** snakebite envenoming, pulmonary embolism, Russell’s viper, D-dimers, CT pulmonary angiography, echocardiogram, thrombosis, anticoagulant therapy

## Abstract

Snakebite envenoming and its resulting complications are serious threats to the health of vulnerable people living in rural areas of developing countries. The knowledge of the heterogeneity of symptoms associated with snakebite envenoming and their management strategies is vital to treat such life-threatening complications to save lives. Russell’s viper envenomation induces a diverse range of clinical manifestations from commonly recognised haemotoxic and local effects to several rare conditions that are often not reported. The lack of awareness about these unusual manifestations can affect prompt diagnosis, appropriate therapeutic approaches, and positive outcomes for patients. Here, we report pulmonary thromboembolism that developed in three patients following Russell’s viper envenomation and demonstrate their common clinical features and diagnostic and therapeutic approaches used. All patients showed clinical signs of local (oedema) and systemic (blood coagulation disturbances) envenomation, which were treated using polyvalent antivenom. They exhibited elevated heart rates, breathlessness, and reduced oxygen saturation, which are non-specific but core parameters in the diagnosis of pulmonary embolism. The recognition of pulmonary embolism was also achieved by an electrocardiogram, which showed sinus tachycardia and computed tomography and echocardiogram scans further confirmed this condition. Anti-coagulant treatment using low-molecular-weight heparin offered clinical benefits in these patients. In summary, this report reinforces the broad spectrum of previously unreported consequences of Russell’s viper envenomation. The constant updating of healthcare professionals and the dissemination of major lessons learned in the clinical management of snakebite envenoming through scientific documentation and educational programs are necessary to mitigate the adverse impacts of venomous snakebites in vulnerable communities.

## 1. Introduction

Snakebite envenoming (SBE) is an acute and serious medical emergency, which induces a diverse range of clinical manifestations including local and systemic effects [1]. For educational, therapeutic, and diagnostic purposes, the pharmacological effects of snake venoms are traditionally classified according to their clinical effects and the mechanisms of action [2]. The major venom toxin classes include serine and metalloproteases (svMPs), phospholipase A_2_ (PLA_2_s), serine proteases (SPs), three-finger toxins (3FTxs), disintegrins, and snaclecs, which are categorised as neurotoxins, haemotoxins, and tissue-damaging toxins [3,4]. In general, simplified clinical pictures and venom protein compositions have been used to explain and guide the treatment of differential clinical manifestations caused by viper and elapid snakebites [5]. For example, venoms from the Viperidae family contain abundant levels of svMPs, SPs, PLA_2_s, and other minor non-enzymatic molecules, such as C-type lectin-related proteins and disintegrins [6]. These venoms are recognised by primarily targeting key processes in the haemostasis that control the blood clotting leading to haemostatic disturbances [7]. Venom-induced haemostasis disorders are complex and multifactorial pathological conditions, with the involvement of different toxin families with diversified mechanisms of action and targets [8]. Serine proteases with thrombin-like activity can cause an imbalance in blood coagulation factors and accelerate the formation of fibrin clots, whereas other venom components (svMPs and PLA_2_s) can impair the blood clotting process through the modulation of platelet function and aggregation, or the breakdown of phospholipids and coagulation factors [9]. svMPs can also lead to vessel damage and bleeding events due to their impact on basement membranes [10]. On the other hand, elapid snake venoms usually show proteomic profiles mainly composed of biomolecules that are affecting the nervous system, including PLA_2_s, and 3FTxs, which are commonly associated with neurotoxic effects [11]. However, despite the usefulness of this generalisation of clinical aspects, several recent reports have drawn attention to specific snake species such as Russell’s viper (*Daboia russelii*) that are capable of inducing unique complications which were poorly reported in the literature [12].

Russell’s viper has a greater impact in India due to the high number of incidents and resulting deaths and disabilities [13]. Proteomic assessments have pointed out the challenging variations in venom composition, which may have implications for the management and therapy of bite victims [14]. Its venom contains the major toxins found in viper venoms, with a predominance of PLA_2_s, svMPs, and Kunitz-type serine protease inhibitors in most venom samples characterised using the venomics approach. Russell’s viper venom is a rich source of bioactive molecules that act on the haemostatic system [15]. As a result, this venom is predominantly haemotoxic with prominent local effects, but it can also cause neurotoxic effects [16] and several different rare conditions such as renal, adrenal, and pituitary haemorrhages, rectus sheath haematoma, priapism, peripheral arterial thrombosis, and splenic rupture [17]. It is critical to report such rare clinical effects to improve the knowledge of healthcare professionals, especially in low-resource settings, to tackle such issues in SBE victims.

Pulmonary thromboembolism is a life-threatening medical condition that requires prompt attention and medical treatment [18]. This cardiopulmonary event is characterised by haemodynamic decompensation due to the formation and migration of blood clots from elsewhere and the blocking of specific blood vessels within the lungs. This impairs efficient gas exchange in the lungs and limits essential blood supply to tissues [18]. The delayed recognition of this condition results in high mortality and morbidity rates [19]. There are several pathological conditions and risk factors associated with this condition such as prolonged inactivity and vasculature damage that can induce deep vein thrombosis and subsequent embolism [20]. Indeed, SBE has been reported to induce pulmonary embolism in a few cases [21,22,23], although Russell’s viper bite-induced thromboembolism has been poorly reported. The non-specific nature of clinical manifestations for pulmonary thromboembolism often affects prompt diagnosis and treatment. Here, we report pulmonary thromboembolism developed in three patients following Russell’s viper bites and demonstrate the clinical symptoms and diagnostic and therapeutic approaches used to tackle this condition. The information provided in this report will increase awareness of healthcare professionals about this condition and management strategies in Russell’s viper (or other snakebite) victims and improve their ability to treat SBE victims effectively.

## 2. Results

### 2.1. Clinical Presentation of Patients

The first patient was a 28-year-old male. At the moment of the bite, he and his brother were sitting in their backyard, and just after he shouted, his brother killed the snake. The offending snake was identified as Russell’s viper by a qualified herpetologist in a local hospital. He was brought to our emergency department complaining of acute breathlessness and collapsed while standing. He lost consciousness for approximately five minutes and was then brought to the emergency room with recovered consciousness. Although he recovered spontaneously, he was extremely weak and dyspnoeic. He was also diaphoretic and tachypnoeic but denied any associated chest pain or palpitations. No tonic-clonic activity was witnessed, and he did not experience incontinence. His medical history was significant for Russell’s viper bite to his left leg, just above the ankle ten days previously. In the previous hospital, he developed swelling up to the left knee and had no other signs of haemorrhage. However, he displayed prolonged clotting time, indicating evidence of systemic envenoming, as noted in his medical records. Both the prothrombin (PT) (14.4 s) and activated partial thromboplastin (aPTT) (36.4 s) times were prolonged and the INR of clotting was 1.13. Hence, he was treated with 20 (200 mL) vials of polyvalent antivenom (VINS bioproducts, Telangana, India) raised against the Indian Big Four snakes (Russell’s viper, cobra, krait, and saw-scaled viper). The patient’s clotting time returned to normal values within 36 h following the first administration of antivenom. He did not have a history of any other significant medical conditions and was not taking any medications or herbal supplements. Upon admission to our emergency room, a physical examination revealed a diaphoretic and dyspnoeic patient without focal neurological findings. His heart rate was regular but tachycardic (128 beats/minute), his blood pressure was 126/72 mmHg without orthostatic changes, and his respiratory rate was 32 breaths/minute. The room air oxygen saturation was 88%, and therefore he was provided with high-flow oxygen through a non-rebreather mask for around 6 h. His arterial blood gas analysis in room air revealed hypoxemia with an elevated alveolar-arterial oxygen gradient. There was no obvious vein distension in the neck, audible murmurs, loud P2, or right ventricular lift. The chest examination revealed reduced breath sounds bilaterally at the lung bases. The findings of heart and abdominal examinations were unremarkable. He had no clinical signs of compartment syndrome. His serum electrolytes, glucose, blood urea, creatinine, complete blood counts, and coagulation parameters were normal. The results of a computed tomographic (CT) scan of his head were negative for bleeding, aneurysm, or an embolic event. His chest X-ray was also clear. 

The second patient was a 19-year-old female who was brought to our emergency department at around 8 pm with a history of snakebite on her right foot while walking in her garden one hour previously. The offending snake was killed and identified as Russell’s viper by a qualified herpetologist. On examination, she was conscious, well-oriented, anxious, afebrile, and haemodynamically stable. Her systemic examination was unremarkable, and local examination revealed swelling of the foot with visible fang marks and palpable tender right-inguinal lymph nodes. She had active bleeding from the bite site and displayed prolonged PT and aPTT. Hence, 10 (100 mL) vials of polyvalent antivenom (Bharat Serums and Vaccines Limited, Maharashtra, India; Batch No: A 1822017; date of expiry: 1 March 2026) raised against the Indian Big Four snakes were administered to normalise the coagulation profile according to the standard protocol. After six hours, her coagulation profile was abnormal (PT test 42.5 s–control–13.5 s, INR–4.44). Hence, she was administered with a further 10 vials of antivenom to completely restore the coagulation parameters. On the third day of her stay in the intensive care unit, she was agitated, hypotensive (63/35 mmHg), tachycardic (123 beats/minute), hypoxemic (80% on room air), and tachypnoeic (50 breaths/minute). The cardiovascular examination was unremarkable except for tachycardia. No signs of deep vein thrombosis were observed using a three-point compression ultrasound scan. The laboratory tests then revealed normal haemoglobin (11.4 g%), platelets (190 × 10^9^/L), and troponin I (0.23 pg/mL) levels. The arterial blood gas analysis was performed with 5 L/min supplementary oxygen, and it showed respiratory alkalosis (pH 7.52), partial pressure of O_2_ as 95 mmHg, partial pressure of CO_2_ as 24 mmHg, HCO_3_ as 22.9 mmol/L, and O_2_ saturation as 99%. 

The third patient was a 45-year-old male who was brought to our emergency room complaining of haemoptysis. The associated symptoms were shortness of breath, exhaustion, chest tightness, and pain in the umbilical region. He was also diaphoretic and tachypnoeic but denied any associated chest pain or palpitations. His medical history was significant for a recent Russell’s viper bite to his right foot eight days previously. He was working in his cattle shed with his coworkers when he was bitten by a Russell’s viper. He was promptly taken to a local hospital, where he received 28 vials of antivenom (Bharat Serums and Vaccines Limited, Maharashtra, India; Batch No: A 18222055; date of expiry: 31 July 2026) to stabilise his coagulation profile. He developed swelling up to the right thigh and had no signs of haemorrhage but displayed prolonged clotting time, indicating evidence of systemic envenoming. Based on the discharge summary, the patient suffered from prolonged coagulopathy. The initial administration of 10 vials of polyvalent antivenom did not result in any improvement, nor did the subsequent 10 vials that were given over 36 h. It was only after another eight vials were administered, that the patient’s condition started to improve. He had no history of any other medical conditions. Upon physical examination in the emergency room, he was acutely ill-looking, tachycardic, and tachypnoeic, with poor distal perfusion and cold extremities. There were no adventitious heart or lung sounds and palpable central and peripheral pulses. His vital signs on arrival were notable for tachycardia (133 beats/minute), tachypnoea (48 breaths/minute), blood pressure (100/50 mmHg), and oxygen saturation (80–86% in room air). His abdominal examinations were unremarkable except for tenderness in the umbilical region. His chest and abdominal X-rays were unremarkable. Point-of-care ultrasonography revealed no ascites, pleural effusion, or pericardial effusion. The haematology screening showed normal haemoglobin levels (13 g%) but a low count of platelets (124 × 10^9^/L) and a high total white blood cell count (15.45 × 10^9^/L).

None of the patients used a tourniquet to the lower limb before hospitalisation. They were not taking any other medications or herbal supplements. They did not display any clinical signs of compartment syndrome. None of the patients had any pre-existing cardiac/pulmonary disease. The laboratory findings confirmed elevated levels of D-dimers in all three patients [first patient: 2236 ng/mL; second patient: 3862 ng/mL; third patient: 1798 ng/mL (normal level should be up to 200 ng/mL)]. The levels of D-dimers were measured on the day the patients were admitted.

### 2.2. Cardiogram Methods and CT Pulmonary Angiogram Confirmed Pulmonary Embolism

**Figure 1:**
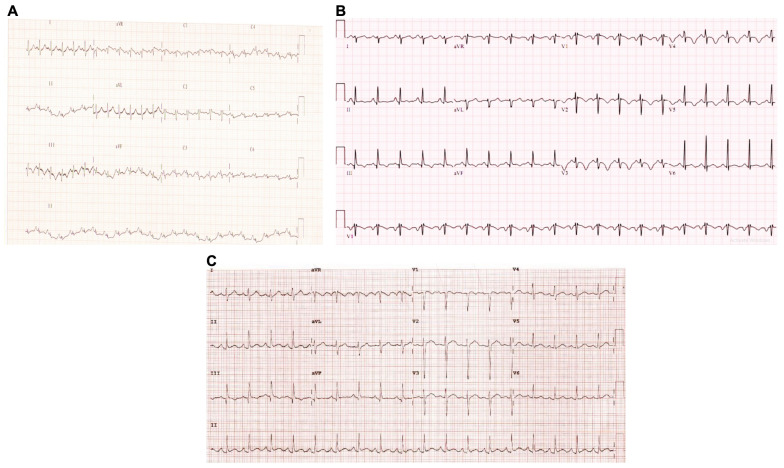
The electrocardiogram reveals sinus tachycardia in Russell’s viper bite patients. The electrocardiogram analysis of patients 1 (**A**), 2 (**B**), and 3 (**C**) is shown. The examination was carried out on the day of admission at Manian Medical Centre.

CT pulmonary angiography is widely recognised as a gold standard approach for diagnosing cases of pulmonary embolism [24]. This imaging technique allows the clear visualization of pulmonary vasculature and clots [25]. However, other tools such as a cardiogram are also valuable in clinical settings despite limited sensitivity [26]. In this report, we illustrated the utility of a combination of these diagnostic tools. All three patients displayed sinus tachycardia on the electrocardiogram with S wave in lead I (S1), Q wave in lead III (Q3), and T wave inversion in lead III (T3) (SIQ3T3 pattern) (Figure 1). 

**Figure 2:**
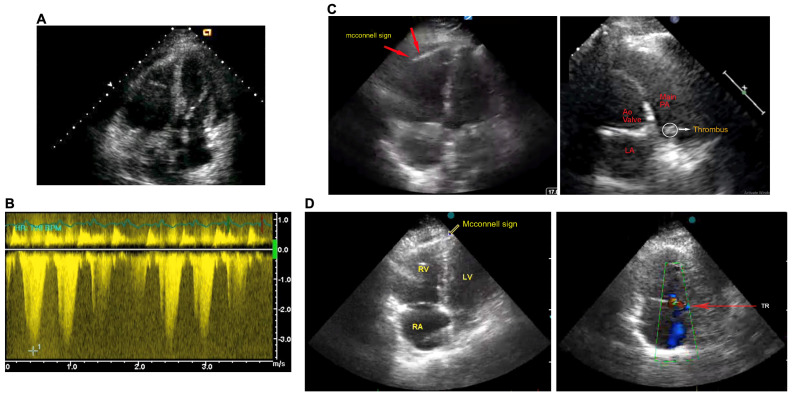
The transthoracic echocardiogram analysis in Russell’s viper bite patients. The echocardiogram was performed on patients 1 (**A**,**B**), 2 (**C**), and 3 (**D**). (**B**) shows pressure in the pulmonary artery. The typical McConnell’s sign is shown in relevant images. RV—right ventricle; LV—left ventricle; RA—right atrium; AO—aortic valve; PA—pulmonary artery; LA—left atrium; TR—thrombus. The examination was carried out on the day of admission at Manian Medical Centre.

In the transthoracic echocardiogram, all patients had right ventricular (RV) hypokinesia without a patent foramen ovale and RV dysfunction, elevated RV pressure, and RV dilatation (Figure 2). Moreover, an atrial or ventricular septal defect with severe pulmonary hypertension was observed in the first patient (Figure 2A). The echocardiography revealed elevated pressure in the pulmonary artery (Figure 2B). Interestingly, the second (Figure 2C) and third (Figure 2D) patients had dilated right atrium and ventricle, with regional wall motion abnormalities at the basal and mid-right ventricular free wall with apical hypercontractility (McConnell’s sign). The thrombus was visualised in the main pulmonary artery of these patients. In all three patients, the presence of the 60/60 sign was seen and pulmonary arterial hypertension was impacted in all of them. 

**Figure 3:**
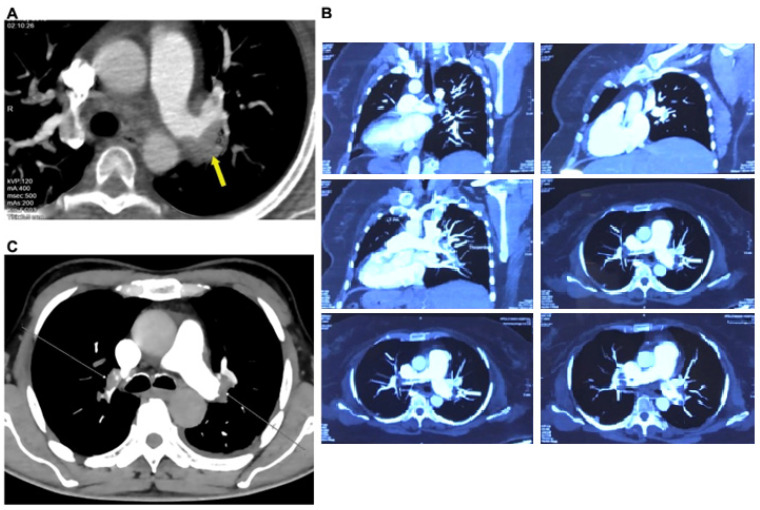
CT pulmonary angiogram in all Russell’s viper bite patients. The diagnosis of pulmonary embolism was achieved by means of CT pulmonary angiography using a contrasting dye in patients 1 (**A**), 2 (**B**), and 3 (**C**). The arrows indicate the thrombi. The examination was carried out on the day of admission at Manian Medical Centre.

In a diagnostic CT pulmonary angiogram, an embolism was detected on the left side in the first patient (Figure 3A) and bilaterally in the second (Figure 3B) and third (Figure 3C) patients. The involvement of the pulmonary trunk, right and left pulmonary arteries, segmental branches, and subsegmental branches in pulmonary embolism was found in the second and third patients. In terms of the lobar distribution of pulmonary emboli, the most commonly involved sites were the lower lobe branches on the right more than the left, followed by the upper lobe branches in the second and third patients. 

### 2.3. LMWH Was Administered to Successfully Resolve PE

All three patients were treated following the current guidelines of the European Society of Cardiology [27] using low-molecular-weight heparin (Enoxaparin 1.0 mg/kg) every 12 h. The first patient received subcutaneous doses of 60 mg two times per day for 10 days and the other two patients were treated for seven days with the same dose. The third patient also received a 10 mg bolus of recombinant tissue plasminogen activator (Actilyse—Alteplase, Boehringer Ingelheim Pharma GmbH & Co. KG, Ingelheim, Germany), followed by another 90 mg intravenous infusion with a syringe pump over 90 min. All three patients recovered well without any sequelae and were discharged from our hospital on day 10. Upon discharge, all three patients took 110 mg of oral dabigatran daily for three months, with their INR monitored monthly. After three months, no other medications were provided but patients continued to follow up for three more months. In addition to antivenom treatment and low-molecular-weight heparin, the patients received intravenous fluids, paracetamol, proton pump inhibitors, and tetanus toxoid during hospitalization as necessary. 

## 3. Discussion

Russell’s viper venom is known to induce haemotoxic, neurotoxic, nephrotoxic, and local envenomation effects [16,28]. However, rare complications such as priapism, adrenal haemorrhage, splenic rupture, rectus sheath haematoma, sialolithiasis, and pseudoaneurysm induced by Russell’s viper envenomation have received wider attention in the literature in recent years [12]. The documentation of such rare clinical presentations has highlighted several previously underreported scenarios that require immediate medical action and provided extensive knowledge for healthcare professionals to support the clinical management of SBE in wider settings. Therefore, it is important to report further rare complications induced by SBE to continue to improve the knowledge and the distinctive diagnostic and therapeutic approaches available to tackle such conditions. Here, we demonstrate the development of pulmonary thromboembolism following Russell’s viper bites in three patients. While this condition developed within three days in one of these patients, it took more than a week after the bite to develop in the others. All patients presented typical clinical features of envenomation by Russell’s viper and were treated with antivenom. Despite the antivenom treatment, they all developed pulmonary thromboembolism. 

The coagulation abnormalities following viper envenomation, including from Russell’s viper, are widely reported [1,16,29]. In line with the previous reports, the impact of Russell’s viper envenomation on blood coagulation is evidenced in all three patients as demonstrated through bleeding and prolonged clotting times. However, the reports on pulmonary embolism following SBE are poorly reported. Some studies have reported the occurrence of pulmonary embolism in severe envenomation by *Crotalus scutulatus* [22], Moroccan viper [23], *Bothrops lanceolatus* [30], and unknown viper species in France [21] and Greece [31]. To our knowledge, this manifestation has not been previously documented in victims following Russell’s viper envenomation. Only a few potential mechanisms for viper venoms to induce this clinical manifestation in SBE victims have been postulated. The direct actions of venom molecules on different stages of the blood coagulation cascades are mainly responsible for inducing hypercoagulability in victims [32]. Venom-induced consumption coagulopathy is a well-known phenomenon in SBE where the venom can induce a rapid consumption of coagulation factors and platelets by inducing clotting, and later resulting in excessive bleeding [33]. Indeed, Russell’s viper venom has been demonstrated to induce peripheral arterial thrombosis in victims, and this was underpinned by its acute effects on platelet activation and plasma/whole blood clotting [34]. This venom contains a series of proteolytic enzymes, particularly thrombin-like enzymes with fibrinolytic properties, and they can activate coagulation disturbances resulting in unwanted clotting or bleeding complications [35]. Moreover, Russell’s viper venom contains factor V [36] and X [37] activators, and they can induce clotting through the conversion of these factors to their active forms. Therefore, it is entirely possible for Russell’s viper venom to induce blood clotting through its acute effects on clotting factors and platelets, and due to the instability of such clots, they could migrate to the pulmonary artery through venous circulation. 

The diagnosis of pulmonary embolism in SBE patients may not be straightforward due to the non-specific nature of the symptoms and their rare occurrence along with the complexity of other envenomation complications. Tachycardia is a valuable clinical indicator that helps in the recognition of pulmonary embolism [38]. This indicative symptom was present in all patients in this study. Despite its diagnostic value, an elevated heart rate is also usually present in other medical disorders. Thus, the investigation and confirmation of suspected pulmonary embolism were clinically supported by a combination of other techniques such as an electrocardiogram, echocardiogram, and CT pulmonary angiography. Sinus tachycardia (heart rhythm abnormality) is one of the most prevalent abnormalities in the context of a pulmonary embolism [38]. This change in the electrical signals in the heart was recorded for all patients. The Wells score is a clinical decision tool commonly used to estimate the risk of a pulmonary embolism that takes into account a series of criteria such as symptoms and risk factors [39]. Finally, imaging techniques for pulmonary embolism, as illustrated in this study using CT pulmonary angiography, have been useful for its clinical assessment.

Antivenoms were used in the management of envenomation in all patients. Not all envenomation complications such as local effects are completely prevented/treated by antivenom, and therefore they require additional treatments [1,40]. To tackle pulmonary embolism, all patients were treated with an anti-coagulant drug (heparin) in this study. One patient had to undergo fibrinolysis using a tissue plasminogen activator. These approaches are key for the treatment of thrombotic events leading to pulmonary embolism of different aetiologies [41,42]. Similar treatments using low-molecular-weight heparin have been used on SBE victims with considerable success, as demonstrated here [21]. However, clinicians should be cautious due to the limited efficacy of anti-coagulant drugs in some regions and the possibility of haemorrhage, particularly during the acute phase of envenomation. The molecular variability in venoms [43] and their diverse mechanisms of toxicity make it difficult to generalise and translate these findings in a wider context for all Russell’s viper bite cases. There could be several other factors including the hypercoagulable nature of patients’ blood due to the increased production of clotting factors and platelets, and prolonged inactivity that could be attributable to this condition [44]. We cannot rule out the indirect effects of antivenom in inducing such complications in SBE victims either. 

## 4. Conclusions

In summary, capacity building among clinicians is a crucial step as a potential mitigation strategy to prevent adverse impacts on SBE victims. This study adds further knowledge on Russell’s viper bite-induced complications with a specific focus on pulmonary embolism by providing insights and useful guidelines for diagnosing and treating this condition in Russell’s viper bite patients. The approaches used here for the diagnosis and treatment of this condition can be adapted for other SBE patients. Our multiple efforts to understand and document the wide spectrum of clinical manifestations induced by Russell’s viper envenomation, including uncommon complications, provide a special opportunity for improving the clinical management of SBE. Collectively, the clinical presentations discussed here add to a series of articles showing that continued attention to different toxicological effects and hospital scenarios following SBE is fundamental for better outcomes. These detailed case studies serve as examples that clearly illustrate how the knowledge and training of healthcare professionals can be critical to public health practice in challenging times, such as the acute nature of SBE.

## 5. Methods

### 5.1. Data Collection 

The patient data were obtained from medical records. Imaging studies were retrieved from the electronic health record (EHR) via patient portals.

### 5.2. D-Dimer Levels

The plasma D-dimer levels were measured using an automated quantitative latex-based, immuno-agglutination assay (HemosIL D-Dimer HS 500, Werfen, Gurgaon, India).

### 5.3. Clotting Tests

Clot-based assays (including PT and aPTT) were photo-optically measured using a single-channel semi-automated Haemostasis Analyzer (Hemostar XF 1.0, Tulip Diagnostics (P) Ltd., Gurgaon, India). Uniplastin and calcium chloride were procured from Tulip Diagnostics.

### 5.4. Echocardiogram 

The transthoracic echocardiography was performed using GE Vivid E9 with a 3.5 MHz probe (GE, Vingmed Ultrasound, Hortom, Norway) by an experienced emergency physician. 

### 5.5. CT Pulmonary Angiogram

The diagnostic CT pulmonary angiography (CTPA) was performed on all three clinically suspected pulmonary embolism patients who had a Wells score of ≥4.5. Following standard procedure, the scans were carried out with a 64-slice helical CT scanner (Aquilion 64TM; Toshiba Medical Systems Corp., Tokyo, Japan) and angiographic contrast material (Omnipaque 350; GE Healthcare, Chicago, IL, USA). The volume of contrast material used was 50 to 70 mL at a rate of 5 mL/s, followed by 20 mL of normal saline at the same rate. Post-processing multiplanar reconstruction was carried out on a dedicated workstation. A well-trained radiologist analysed the data and other radiological abnormalities for all patients. 

## Data Availability

All data from this study are included in this article.
